# Differentiating weight-restored anorexia nervosa and body dysmorphic disorder using neuroimaging and psychometric markers

**DOI:** 10.1371/journal.pone.0213974

**Published:** 2019-05-06

**Authors:** Don A. Vaughn, Wesley T. Kerr, Teena D. Moody, Gigi K. Cheng, Francesca Morfini, Aifeng Zhang, Alex D. Leow, Michael A. Strober, Mark S. Cohen, Jamie D. Feusner

**Affiliations:** 1 Semel Institute for Neuroscience and Human Behavior, University of California Los Angeles, Los Angeles, California, United States of America; 2 David Geffen School of Medicine, University of California Los Angeles, Los Angeles, California, United States of America; 3 Department of Biomathematics, University of California Los Angeles, Los Angeles, California, United States of America; 4 Department of Internal Medicine, Eisenhower Medical Center, Rancho Mirage, California, United States of America; 5 Department of Psychology, Harvard University, Cambridge, Massachusetts, United States of America; 6 Department of Psychiatry, University of Illinois College of Medicine, Chicago, Illinois, United States of America; 7 Departments of Neurology, Radiology, Biomedical Physics, Psychology, Bioengineering and California Nanosystems Institute, University of California Los Angeles, Los Angeles, California, United States of America; Universita degli Studi di Udine, ITALY

## Abstract

Anorexia nervosa (AN) and body dysmorphic disorder (BDD) are potentially life-threatening conditions whose partially overlapping phenomenology—distorted perception of appearance, obsessions/compulsions, and limited insight—can make diagnostic distinction difficult in some cases. Accurate diagnosis is crucial, as the effective treatments for AN and BDD differ. To improve diagnostic accuracy and clarify the contributions of each of the multiple underlying factors, we developed a two-stage machine learning model that uses multimodal, neurobiology-based, and symptom-based quantitative data as features: task-based functional magnetic resonance imaging data using body visual stimuli, graph theory metrics of white matter connectivity from diffusor tensor imaging, and anxiety, depression, and insight psychometric scores. In a sample of unmedicated adults with BDD (n = 29), unmedicated adults with weight-restored AN (n = 24), and healthy controls (n = 31), the resulting model labeled individuals with an accuracy of 76%, significantly better than the chance accuracy of 35% ($\hat{p}<10^{‑4}$). In the multivariate model, reduced white matter global efficiency and better insight were associated more with AN than with BDD. These results improve our understanding of the relative contributions of the neurobiological characteristics and symptoms of these disorders. Moreover, this approach has the potential to aid clinicians in diagnosis, thereby leading to more tailored therapy.

## Introduction

Anorexia nervosa (AN) and body dysmorphic disorder (BDD) are psychiatric disorders that share several phenomenological characteristics such as distorted perception of appearance [1], obsessive and compulsive symptoms [2], and poor insight [3,4]. BDD is characterized principally by an individual’s preoccupation with a perceived defect in their appearance [1]. Prevalence in the general population is estimated between 0.7% [5,6] and 2.9% [7,8]. AN is characterized by restriction of energy intake, fear of gaining weight or becoming fat, disturbance in the individual’s perception of body weight, and significantly low BMI [1]. The prevalence is estimated to be 0.9% and 0.3% in females and males, respectively [9].

Both disorders are very serious conditions that confer an elevated risk of mortality. Of all psychiatric disorders, AN has the highest risk of mortality: 0.6% per year [10,11]. Medical morbidity and mortality related to the starvation state are major risks in affected persons [12]. During the lifetime of an individual with a BDD diagnosis, 24–28% will attempt suicide [13]; the suicide-related mortality rate in individuals with BDD is roughly 0.3% per year [14]. The alarmingly frequent mortalities and severe risks associated with AN and BDD underscore that it is imperative to recognize these disorders and to intervene with treatment to minimize poor outcomes.

The selection of an effective treatment for AN or BDD is aided by proper diagnosis. Cognitive behavioral therapy (CBT) can be effective in BDD [15], but its usefulness in AN is minimal [16]. BDD can respond to treatment with selective serotonin reuptake inhibitors (SSRIs), but evidence for this in AN is lacking [17,18]. Family-based psychotherapy in adolescents with AN has been shown to be effective [19].

Clinically, it can be difficult in some cases to distinguish between AN and BDD. Similarities in symptom characteristics are such that some researchers have raised the question of whether one disorder should be considered a subtype of the other, or at least have noted that they share pathological features [20,21]. For example, 30% of individuals with BDD have significant weight-related appearance concerns (e.g., concern about their cheeks being too fat), even if they do not have a comorbid eating disorder [22]. Consequently, individuals with BDD may restrict calorie intake and engage in appearance-related compulsive behaviors. In this example, Diagnostic Statistical Manual (DSM-5 or DSM-IV) criteria would categorize the person as having AN or AN plus BDD if the body weight was less than 85% of that expected, and BDD alone if 85% or above. However, the behavioral manifestations and subjective experience for the patient may be otherwise identical.

In fact, individuals frequently meet criteria for both AN and BDD [23,24]. Of those with AN, 25–39% are diagnosed with lifetime BDD; 32% of those with BDD will have a lifetime eating disorder [23–25]. In studies that directly compared individuals with AN to those with BDD, the two groups shared lower body satisfaction, lower appearance evaluation, lower attitudes toward one’s self, more body areas of dissatisfaction, and greater preoccupation with being overweight, compared with healthy controls [26–28]. In terms of clinically distinguishing features, aside from the low body weight criteria in AN, the sex distribution is notably more skewed toward females in AN (90% female), whereas BDD has a more equal prevalence across sexes [7,29]. While insight tends to be poor in both conditions, it is worse on average in BDD [4]. With these data in mind, we believe that it would be advantageous to have additional quantitative means of distinguishing between these conditions.

Neuroimaging has provided several features that may help distinguish between individuals with AN, those with BDD, and healthy controls. Recent research suggests that distorted perceptions of appearance may be linked to abnormalities in the visual system [30–32]. A combined analysis of data from task-based functional magnetic resonance imaging (fMRI) and EEG showed that, relative to healthy controls, individuals with AN and those with BDD exhibited hypoactivity in both early secondary visual regions and the dorsal stream when viewing low-spatial frequency face and house stimuli [33]. An event-related potential (ERP) analysis of the same EEG data likewise revealed that the amplitude of P100 responses was smaller in individuals with AN than in those with BDD and in healthy controls, whereas N170 latencies were longer and N170 amplitudes were smaller in AN and BDD compared with healthy controls [34].

Diffusion tensor imaging (DTI) additionally has demonstrated that brain white matter connectivity patterns differ in individuals with BDD and healthy controls [35], and between individuals with AN and healthy controls [36]. Connectivity pattern abnormalities in white matter networks have been found within the visual system of individuals with BDD [3,37]. Among the few studies that have compared AN and BDD directly, correlations were present between psychometric insight and ERP amplitude in BDD but not in AN [34]. Insight was correlated with the network measure path length in white matter in AN but not in BDD [3]. Despite these known neurological differences, a differential diagnosis currently is made using criteria based principally on weight, observed behaviors, and reported symptoms.

It is important also to distinguish psychiatrically ill individuals from healthy controls. This is an issue particularly in adolescents, the age group most likely to develop AN and BDD. The role of clinicians is to identify patients who would benefit from treatment while avoiding a pathologic diagnosis for normal adolescents. Mislabeling developmentally-normal, self-conscious behavior (e.g., overvaluing appearance, mirror-checking) as a disorder can have detrimental effects on an individual's perceived identity (Rosenhan, 1973). On the other hand, mislabeling a disorder as normal development could normalize the pathologic behaviors and experiences of AN and BDD (e.g., purging, compulsive behaviors, significant interference in functioning, emotional distress). BDD often goes undetected in adolescents [38], yet the suicide attempt rate is possibly higher than in adults (21–44%) [39].

With this background in mind, our current study had two goals: 1) to understand mechanistically the multivariate differences in neurobiology and psychometric scales that map to the diagnoses, and 2) to aid clinicians by developing an enhanced and more objective means of distinguishing between AN, BDD, and healthy controls. Both of these goals were achieved by combining multimodal neuroimaging and psychometric data into a multivariate logistic regression model. The feature weights of the multivariate model address the first goal by providing an evidence-based magnitude, direction, and significance as to how each modality relates to a particular diagnosis. The model’s probabilistic outputs address the second goal by providing diagnostic predictions. Although many studies of psychiatric populations collect more than one modality of neurobiological data and multiple psychometric scales, few have combined these in fully integrated models to enhance the accuracy of predictive classification (or for other purposes).

## Materials and methods

### Participants

Our study included data from 84 participants (age 22 +/- 5 years, 88% female): 24 diagnosed with weight-restored AN, 29 diagnosed with BDD, and 31 healthy controls. All participants were unmedicated. The University of California, Los Angeles (UCLA) Institutional Review Board approved the protocol, and all participants provided written informed consent. Capacity to provide informed consent was determined by the study physician (Dr. Feusner). For minors who participated we obtained signed parental permission and youth assent.

For semantic ease, we defined AN/BDD to be all of the participants with either AN or BDD; AN/BDD does not indicate that the participants were comorbid with both disorders, as participants with both AN and BDD were excluded. Participants were of equivalent age and gender (Table 1).

**Table 1:** Demographics.

	AN	BDD	CTL	Test statistic	*p*
**Age (yrs)**	21 ± 5	23 ± 5	21 ± 5	*F* = 2.2, df = 2	0.12
**Female**	23/24 (96%)	26/29 (90%)	25/31 (81%)	𝜒^2^ = 3.1, df = 2	0.21
**BMI**	20 ± 2	22 ± 3	22 ± 3	*F* = 4.1, df = 2	0.02
**Illness duration (months)**	72 ± 63	118 ± 70	N/A	*t* = 2.6, df = 51	0.03
**Lowest lifetime BMI**	16 ± 2	N/A	N/A	N/A	N/A
**Number of participants**	24	29	31	N/A	N/A

Healthy controls (denoted as CTL in this table), participants with AN, and participants with BDD were matched across age, gender, and body mass index (BMI). Participant age and the proportion of females were not significantly different across groups; BMI and illness duration differed significantly between groups. Errors are standard deviation.

We excluded participants who met any of the following criteria: (1) medical disorders affecting cerebral metabolism (e.g., diabetes mellitus or hyperthyroidism); (2) pregnancy; (3) neurological disorders; (4) concurrent Axis I disorders other than dysthymia, major depressive disorder, or generalized anxiety disorder; (5) score >44 (very severe) on the Montgomery-Åsberg Depression Rating Scale (MADRS) [40] and/or major depressive disorder with psychotic features; (6) an assessment of imminent suicidality (by Dr. Feusner); (7) current or past diagnosis of AN (in the BDD group) or a current or past diagnosis of BDD (in the AN group); and (8) current treatment with CBT. Participants were required to be free from psychoactive medications for at least 8 weeks prior to entering the study. To avoid the confounds of acute starvation on brain structure and function, all participants with AN had to be weight-restored during data collection (as defined by BMI ≥ 18.5) despite having previously met full DSM-IV criteria for AN. BDD participants had to score ≥ 20 on the BDD version of the Yale-Brown Obsessive Compulsive Scale (BDD-YBOCS) [41].

### Psychometrics

To establish diagnoses, all participants were administered the Mini International Neuropsychiatric Interview (MINI) [42] and the BDD Diagnostic Module [43], modeled after the DSM-IV. Symptom severity was quantified in AN with modified versions of the Yale-Brown-Cornell Eating Disorder Scale (YBC-EDS) [44] and the Eating Disorder Evaluation (EDE) Edition 16.0D [45], and in BDD with the BDD-YBOCS [41]. Participants with AN and those with BDD, were administered the Brown Assessment of Beliefs Scale (BABS) [46], the Hamilton Anxiety Rating Scale (HAM-A) [47], and the Montgomery-Asberg Depression Scale (MADRS) [40].

### Overview and rationale of feature selection

The principal goal of the study was to create a classification model to help distinguish AN/BDD from healthy controls, and AN from BDD. To make use of a range of neurobiological and psychometric data, we chose structural (DTI) and functional (fMRI) data (acquired in all groups), psychometric ratings measuring anxiety and depression (acquired in all groups), and insight (acquired in AN and BDD). From the DTI data, we chose to use the graph theory metric of *normalized path length* (NPL) because 1) this global network connectivity measure provides a single summarized metric of white matter network connectivity for each participant, and 2) our previous univariate analysis suggested differing patterns in AN and BDD [3]. We chose to include fMRI task data from brain responses to both low and high spatial frequency bodies and faces. We hypothesized that bodies and faces (rather than the third stimuli class, houses) would best distinguish both controls from AN/BDD and distinguish AN from BDD, because bodies were likely to be more salient for people with AN and faces for those with BDD [48].

### fMRI data

We collected fMRI data as participants engaged in simple tasks of matching photographs of others’ bodies and of others’ faces [49]. Each run contained blocks of images with low-only, normal, or high-only spatial-frequency content, and a control task of matching shapes. Previous analyses suggested that the stimuli types that showed the least overlap between participants with AN/BDD and controls were low and high spatial-frequency images; thus, we used responses to high and low spatial-frequency but not normal spatial-frequency images from the bodies and faces data as features in our model. In the interest of deriving a signal metric per network, we executed a network coherence analysis of the functional data. Based on previous results, we hypothesized that the most informative coherence values would come from three networks of interest: primary visual, higher order visual, and salience networks [50]. For further detail, see *supplementary material*.

### DTI data

The neuroimaging data included DTI in the form of 64 gradient direction diffusion-weighted images with b = 1000 s/mm^2^ and one minimally diffusion-weighted scan (the B_0_ image). Graph theory metric data were obtained from a processing pipeline previously described [3]. Briefly, we derived 87 cortical and subcortical structures using Freesurfer (Martinos Center for Biomedical Imaging, USA) from each participant’s T1-weighted scan data, and we performed continuous fiber tracking from the diffusion-weighted data using DTIStudio software (http://www.mristudio.org). Combining the parcellation and fiber tractography results yielded an 87 x 87 connectivity matrix of tractography relationships between regions. For each participant's data, we calculated the shortest path length between each pair of nodes and then averaged this path length over all nodes to determine the characteristic path length (CPL). We calculated the normalized path length (NPL) as the ratio of observed CPL to the CPL of an identically sized but randomly connected network [51].

### Handling the missing data

As is a challenge with many multimodal datasets, we had missing data for some participants across psychometric and neuroimaging modalities. We addressed this problem using multiple imputation [52]. Due to a modified initial study design, the first eight participants with AN did not complete the BABS. None of the control participants completed the BABS because they, by definition, had no disorder for which they could provide insight. We did not use functional data from 27 of the participants because of their excessive head movement. Of our 84 participants, we had data for 100% of HAM-A/MADRS, 85% (45/53) of the BABS, 100% of DTI, and 68% (57/84) of fMRI.

Additionally, we also performed a complete case analysis using the 57 participants for whom we had no missing data (17 AN, 19 BDD, 21 control). The complete case results—in terms of model performance, feature significance, and feature magnitude—were not dissimilar from the imputation model and thus are not discussed further.

### Diagnostic statistical modeling

We predicted the diagnoses using a two-step multiple-imputation logistic regression model; we first predicted whether a participant was a healthy control or had AN/BDD, and for the latter group, we predicted the disorder (AN or BDD). As features, we used coherence values from fMRI data in primary visual, higher order visual, and salience networks using body and face high- and low spatial-frequency visual stimuli; white matter NPL from DTI data; and anxiety, depression, and insight psychometric scores. We trained these models using cyclical leave-one-out cross-validation and assessed their statistical significance via permutation testing, which yielded an empirical, two-tailed p-value indicated by $\hat{p}$. All confidence intervals (CI) are 95% standard error of the mean (SEM), unless otherwise stated.

## Results

### Dataset information

**Fig 1:**
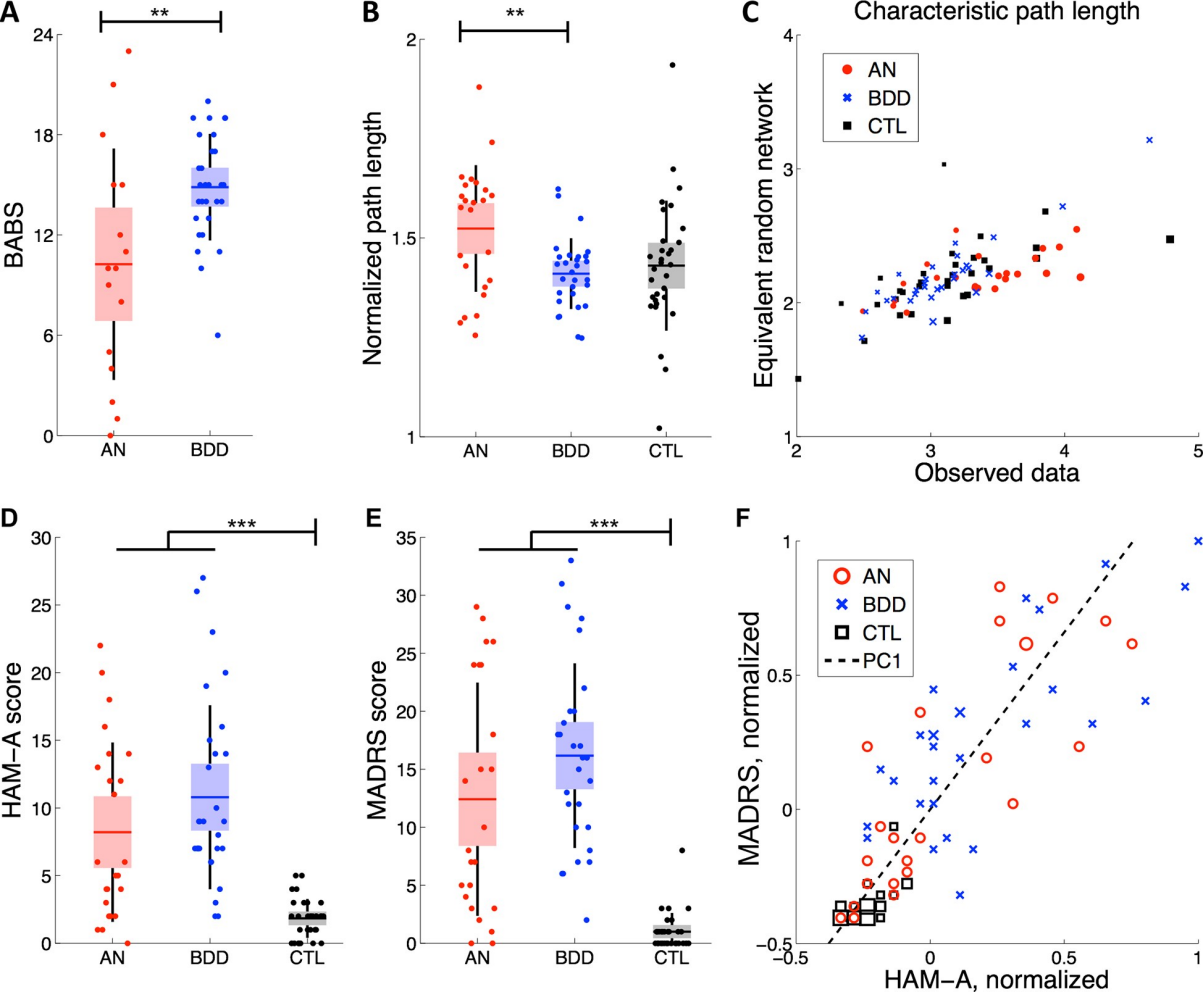
Dataset information. (A) Participants with BDD had significantly different BABS scores than those with AN (*p* = 0.004). In this and all remaining panels, the horizontal line is the mean, box is the SEM 95% CI, black lines demarcate one standard deviation, and dots reflect individual participants. **(B)** NPL differed significantly between AN and BDD groups (*p* = 0.002). **(C)** The NPL comprised the observed CPL and the CPL of an equivalently-sized random network. This plot demonstrates that the denominator of NPL—the CPL of an equivalent random network—does not show large fluctuations that would explain the bimodal distribution in AN as presented in panel B. (D) Participants with AN/BDD scored significantly higher on the HAM-A scale than healthy controls (*p*< 0.001). (E) Participants with AN/BDD scored significantly higher on the MADRS scale than healthy controls (*p*< 0.001). (F) The first single principal component (PC1) of HAM-A and MADRS explained 93% of the variance among all participants with HAM-A and MADRS scores (n = 84). Larger markers represent the presence of more than one participant. BABS = Brown Assessment of Beliefs Scale; HAM-A = Hamilton Anxiety Rating Scale; MADRS = Montgomery-Asberg Depression Scale.

There were significant univariate differences between participants with AN, participants with BDD, and healthy controls. Participants with BDD had significantly higher BABS scores (4.6 ± 1.5) than participants with AN (*t* = 3.1, df = 43, *p* = 0.004) (Fig 1A). Participants with AN, those with BDD, and healthy controls had unequal mean NPL (*F* = 4.8, df = 2, *p* = 0.01, Fig 1B). This difference in central tendency was driven by the AN group (*μ*_AN_-*μ*_BDD_ = 0.11 ± 0.03, *t* = 3.3, df = 51, *p* = 0.002), specifically a bimodal distribution of AN participants with NPLs that were either similar or much longer than the BDD and control groups. Note that this bimodality was not explained by large fluctuations in the denominator (CPL of an equivalent random network) (Fig 1C). Healthy controls scored significantly lower than participants with AN/BDD on both the HAM-A (*μ*_A/B_-*μ*_C_ = 7.8 ± 1.2, *t* = 6.3, df = 82, *p*<10^−4^, Fig 1D) and MADRS (*μ*_A/B_-*μ*_C_ = 13.5 ± 1.6, *t* = 8.2, df = 82, *p*<10^−4^, Fig 1E). We observed a high Pearson correlation between the HAM-A and MADRS scores of all three participant groups (r = 0.85 ± 0.05). Thus, we collapsed both scales into a single combined metric, MH, by extracting the first principal component, which explained 93% of the variance in the data (Fig 1F).

Because of excessive head movement, we had to exclude fMRI data from 29% (7/24) of participants diagnosed with AN, 34% (10/29) of participants diagnosed with BDD, and 32% (10/31) of controls. There was no significant difference in the proportion with missing data across the three groups (Chi-squared, 0.17, df = 3, p = 0.92).

Given that only 68% of coherence values were available, we conducted a preliminary analysis to evaluate whether imputed coherence explained a significant amount of the residual variance in the models. We used a regularized logistic regression model to evaluate the marginal effect of including a combination of high and low spatial frequency stimuli of faces and bodies for each of the three networks. The regularized model did not significantly explain more variation in the data, when considering the number of variables added (deviance difference 12.8, df = 12, p = 0.3). Additionally, the overall accuracy of this overall model decreased with this additional information, likely due to overfitting.

This suggests that the variation accounted for by coherence was explained by other variables in the limited model, and thus, the inclusion of coherence did not improve our prediction of the outcome. Given this result, all subsequent analyses excluded coherence as a feature.

### Distinguishing healthy controls from AN/BDD

**Fig 2:**
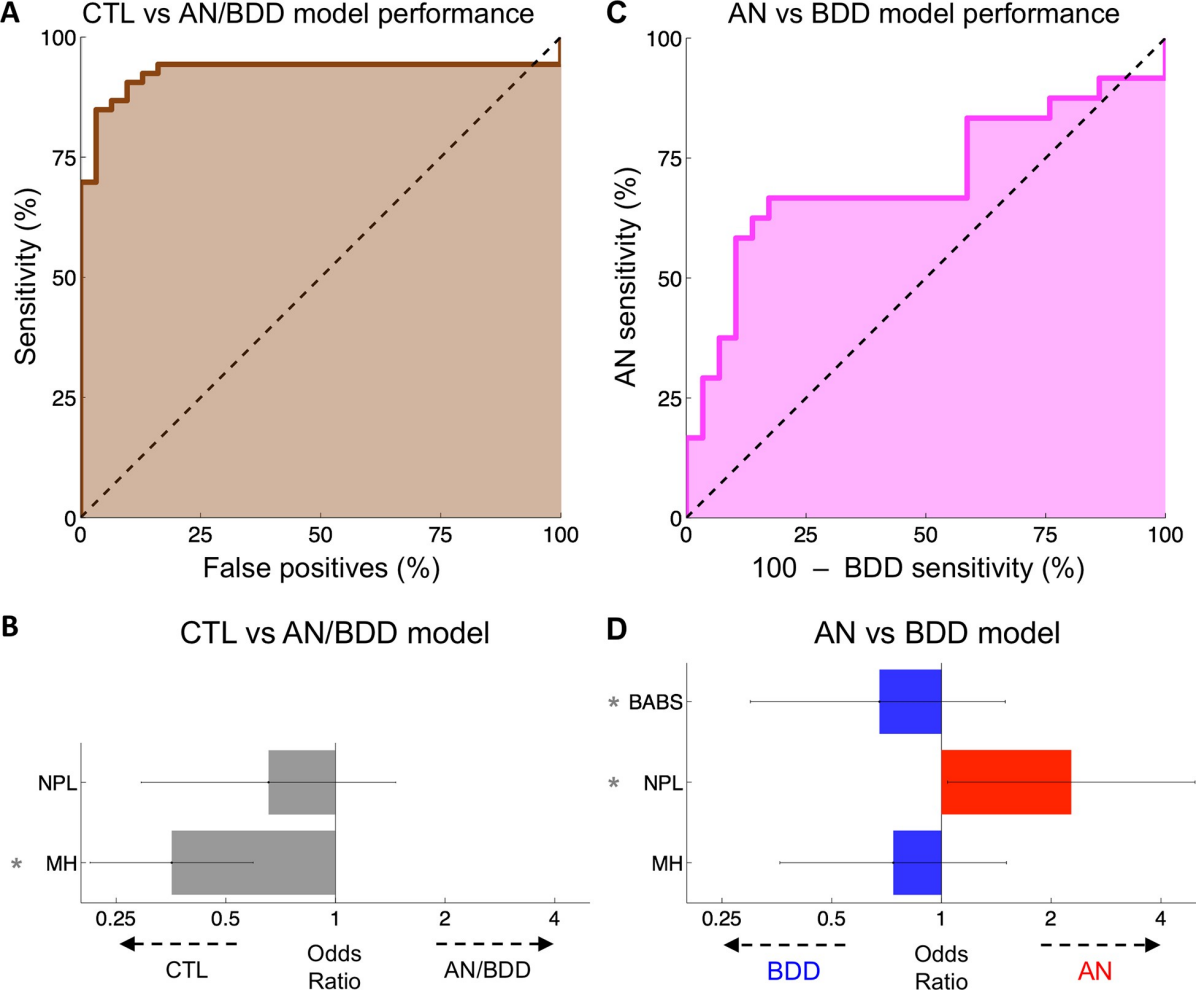
Classifier performance. **(A)** The receiver operator characteristic of the healthy controls (denoted as CTL) vs AN/BDD model. Our model distinguished healthy controls from participants with AN or BDD with an AUC-ROC of 93%. **(B)** Bars reflect the weight of each factor used in the CTL vs AN/BDD classification, expressed as the odds ratio per standard deviation. Error bars are SEM and asterisks demarcate statistically significant features ($\hat{p}<0.05$). **(C)** Our AN vs BDD model distinguished participants with AN from those with BDD with an AUC-ROC of 67%. **(D)** The weight of each factor used in the AN vs BDD classification, expressed as an odds ratio per standard deviation. Error bars are (Gaussian) SEM and thus for reference only; significance values were calculated from the (non-Gaussian) permutations.

When distinguishing healthy controls from participants with AN or BDD, the first step predictive model had a leave-one-out accuracy of 89% (75/84, permutation mean 41%, $\hat{p}<0.01$), a positive predictive value (PPV) of 92% (48/52, permutation mean 53%, $\hat{p}<0.01$), a negative predictive value (NPV) of 84% (27/32, permutation mean 33%, $\hat{p}<0.01$), and an area under the receiver operator characteristic (AUC-ROC) of 93% (Fig 2A). The overall deviance difference was 66.2 (df = 2, p<10^−4^), and this was driven by a significant association of lower MH with healthy controls (estimate 11.82 per PCA-unit, SE 3.00, p<10^−4^, Fig 2B). NPL was not associated significantly with either group (estimate -2.5 per unit, SE 2.5, $\hat{p}$ = 0.31).

### AN vs BDD prediction

The second step predictive model—designed to distinguish participants with AN from those with BDD—had a leave-one-out accuracy of 74% (39/53, permutation mean 40%, $\hat{p}<0.001$), an AN predictive value of 78% (14/18, permutation mean 40%, $\hat{p}<0.01$), a BDD predictive value of 71% (25/35, permutation mean 41%, $\hat{p}<0.001$), and an AUC-ROC of 67% (Fig 2C). The overall difference in deviance was 10.0 (df 3, $\hat{p}$ = 0.006) with a positive association of higher NPL (lower globally efficient white matter connectivity) with AN (log odds 6.0 per unit, SE 2.9, $\hat{p}$ = 0.02), a positive association of higher BABS scores (lower insight) with BDD (log odds -0.07 per unit, SE 0.07, $\hat{p}$ = 0.011 with a non-Gaussian distribution), and no significant association of MH with either group (-0.63 per unit, SE 0.75, $\hat{p}$ = 0.40) (Fig 2D).

### Multistep prediction

The two-step combination of the above models had a three-group leave-one-out accuracy of 76% (64/84, 95% CI: 67–85%, permutation mean 35%, $\hat{p}<10^{‑4}$) with AN predictive value of 63% (12/19, permutation mean 40%, $\hat{p}<0.001$), BDD predictive value of 76% (25/33, permutation mean 41%, $\hat{p}<0.001$), and healthy control predictive value of 84% (27/32, permutation mean 33%, $\hat{p}<0.001$).

## Discussion

By combining multimodal neuroimaging and psychometric data, we were able to differentiate accurately between healthy controls, participants with weight-restored AN, and participants with BDD. This represents the first study to apply this approach to AN and BDD for classification purposes and to understand relative contributions of factors for distinguishing these DSM-defined syndromes. These results shed light on a more dimensional understanding of the neurobiological and psychometric variables that contribute to these disorders.

### Classification performance

While anxiety and depression scores were sufficient to distinguish healthy controls from those with AN/BDD, a combination of psychometric and neuroimaging data best distinguished individuals with weight-restored AN from those BDD. Yet, even with more predictive features, our model distinguishing weight-restored AN from BDD was less accurate than our model distinguishing AN/BDD from healthy controls. As observed clinically, AN and BDD show phenomenological overlap; thus, it is not surprising that differentiating healthy controls from participants with either disorder was much more accurate than distinguishing participants with weight-restored AN from those with BDD. Even though we excluded participants with a DSM diagnosis of both conditions, our lower classification accuracy between weight-restored AN and BDD, as compared to AN/BDD and healthy controls, supports previous findings that some individuals with AN may share symptomatic and behavioral elements of BDD, and vice versa [26–28]. The predictive power our model achieved in distinguishing two phenomenologically overlapping disorders highlights the utility of integrating functional and structural neurobiological data with clinical symptoms and behavior-based data.

Our two-way classification accuracies—89% in distinguishing controls from individuals with AN/BDD, and 74% in differentiating weight-restored AN from BDD—are similar to previous works in other disorders. Using neuroimaging data, other groups have distinguished healthy controls from individuals with social anxiety disorder [53], ADHD [54], major depressive disorder [55], autism [56], and Alzheimer's disease [57] with an accuracy of 80–90%. Fewer groups, however, have attempted to separate individuals with related but distinct diagnoses. The closest comparable studies classified disorder subtypes or predicted the progression of a disorder. For example, functional connectivity distinguished subtypes of ADHD with 65% accuracy [54], regional white matter volume discriminated between treatment-resistant and treatment-sensitive depression with an accuracy of 66% [55], and fMRI and PET data from individuals with mild cognitive impairment predicted who would develop Alzheimer’s disease within 18 months and who would not with 68% accuracy [57].

**Fig 3:**
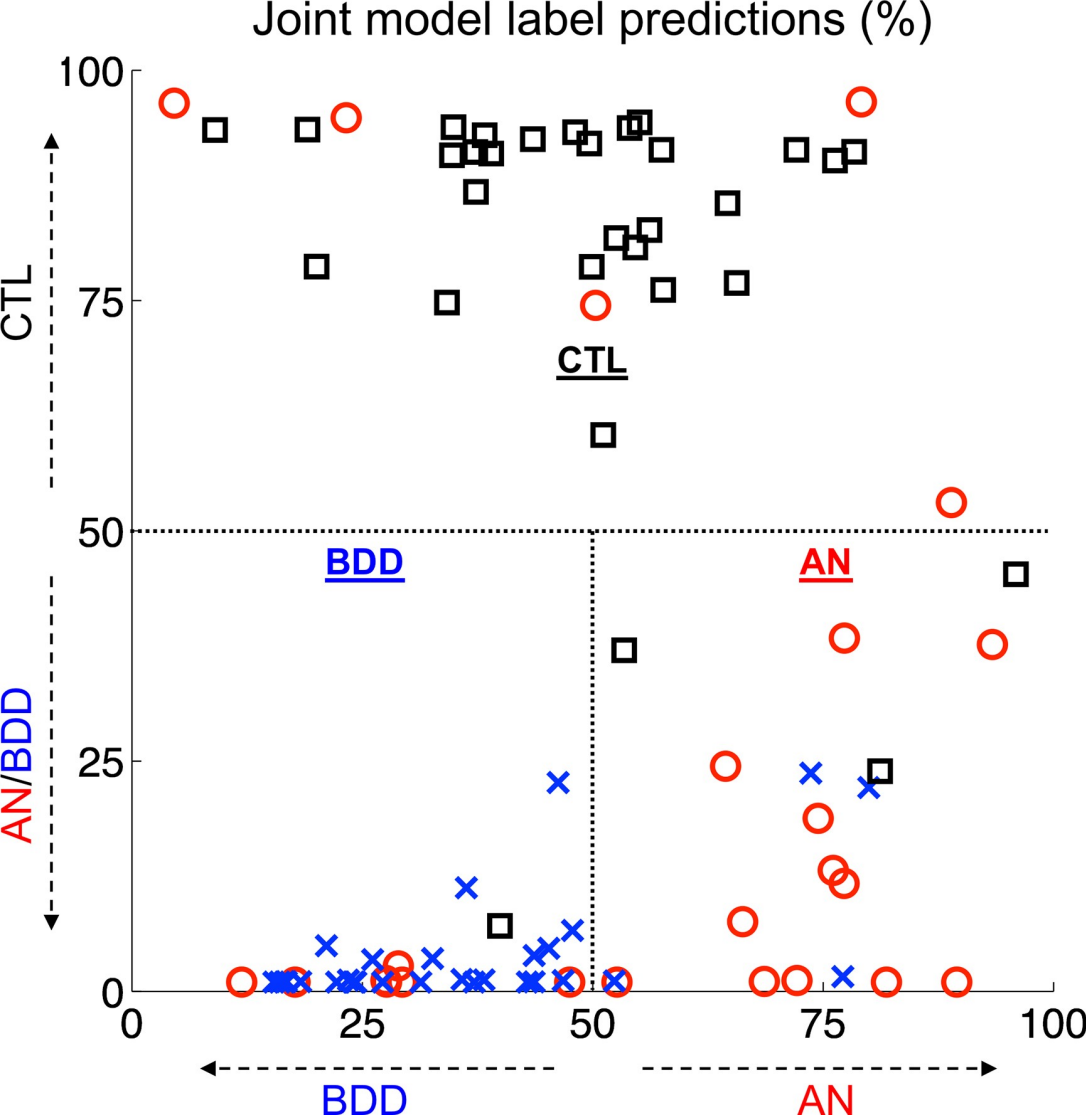
Joint predictions of both models. The synthesis of the control (CTL) vs AN/BDD model and the AN vs BDD model reveals a relatively clear delineation between healthy control participants (black), participants with AN (red), and participants with BDD (blue).

Combining both classification models yields a two-step clinical diagnostic flowchart for a potential patient: (1) Does this individual potentially have an appearance-related psychiatric disorder? (2) If so, how strongly does the evidence suggest that the individual has symptoms of AN or BDD? By combining our participants’ results for both models, the relationship among these conditions is more clear (Fig 3).

There was fairly strong separability between groups. Although the classifier confused some healthy controls and participants with weight-restored AN, none of the participants with BDD were misclassified as healthy controls. This suggests that if the two-stage classifier were to classify an individual as likely having AN/BDD, and then were to assign a final prediction of BDD, there would be a high likelihood that the individual has an actual clinical diagnosis of BDD.

### Neurobiological interpretations

In our model, lower insight contributed significantly to the distinction between BDD and AN. This accords with a previous study that found that, on average, individuals with BDD have lower insight than individuals with AN [4]. Clinical experience also suggests that in AN, more frequently than in BDD, a greater proportion of individuals possess the insight that their body weight is low, yet they nevertheless desire to remain in an underweight state or achieve an even lower weight, accounting for many recalcitrant cases [58,59]. Individuals with AN have compulsive regimens of exercise, food restriction, or selectivity that remain ego-syntonic.

The result that the white matter network connectivity measure (NPL) contributed significantly to the distinction between AN and BDD is in line with our previous finding of higher NPL in AN than in BDD [3]. Our related previous research, however, was univariate, single-modal, and contained no predictive modeling. In other words, whereas our previous univariate analyses could distinguish significant differences among AN, BDD, and healthy controls for each of the neuroimaging and psychometric data separately, the current results provide information about how each measure contributes to differentiating the disorders when accounting for the effects of the others. In the multimodal approach we developed here, insight and NPL both contributed significantly to distinguishing AN from BDD in a multivariate context, prompting the question of how these features might interact with each other to contribute to brain-behavior relationships.

Indeed, the phenomenon of poor insight may itself be linked to NPL in AN. In prior work, we found that poor insight in AN was associated with longer NPL in the anterior and posterior cingulate cortices [3]. Because this finding was specific to these cingulate subregions, it seems to support a relationship between insight and information transfer between these regions (involved in error-detection and conflict monitoring [60]) and the rest of the brain, rather than global network integration. Future studies are warranted to uncover potential relationships between insight and regional or global brain network topology. This will help clarify underlying connectivity patterns associated with the phenomenon of insight, which has important clinical implications for if, and how, patients engage in treatment.

Interestingly, NPL in the AN group demonstrated properties of a bimodal distribution; one subgroup had a clustering of NPL values that resembled those of the BDD and healthy control cohorts, while another subgroup showed much longer NPL values. Thus, longer average NPL, which suggests a less globally-integrated white matter connectivity network across the brain, may be a useful distinguishing feature that separates AN from BDD. The differing network topology of a subgroup of AN raises the question of whether classification methods alternative to DSM might better capture natural “cut-points” of the neuropathology of AN. This was outside of the focus and scope of the present work, and future studies are required to investigate this further.

Our results show that healthy controls could be differentiated easily from individuals with appearance-related psychiatric disorders using a single psychometric metric of anxiety/depression. While this is informative, it illustrates that differentiating healthy controls from weight-restored AN or BDD can be ascertained readily by a clinical assessment; neurobiological data from neuroimaging is unnecessary. The more difficult distinction, in some clinical cases, is between AN and BDD; our model also performed well with this classification.

### Limitations and future directions

In this study, we based classification accuracy results on DSM criteria, though we acknowledge the ongoing debate about the utility of DSM or International Classification of Diseases (ICD) versus dimensional approaches, as suggested by the NIH Research Domain Criteria (RDoC), in the classification of psychiatric disorders. The benefit of developing more advanced algorithms for classification is predicated on the existence of at least some applications for diagnostic classification. Despite limitations of DSM- or ICD-defined diagnostic categories, there is practical value in distinguishing the clinical syndromes of AN from BDD, namely, for the sake of clinical decisions about specific pharmacotherapy and psychotherapy approaches. As outlined above, these are notably different for the diagnostic categories of AN and BDD, and—amongst other important implications of these distinctions—early interventions to prevent the starvation state are imperative for the prevention of medical morbidity and mortality.

On an individual level, our statistical classification approach yielded different results from the gold-standard (clinical diagnoses) in several cases. While this may reflect a limitation of the present method, it could also indicate different interpretations of clinical criteria when applied to specific patients. With a modest sample of the present size, it would not likely be informative to evaluate the classification differences on individuals, but future work may want to consider whether such differences arise from factors that may ultimately prove useful in interventions. There also may be yet-unidentified methodological and clinical variables that moderate our classifier’s performance.

Our classification model was developed with information from participants from the local community. Variations in methods of test administration, neuroimaging, clinical diagnostics, and underlying heterogeneity of these disorders may differ slightly from location to location, so these results need to be further validated on larger, independent datasets from different regions that capture the full range of the disease before direct application to clinical medicine.

Prior to any considerations of applying this method in clinical diagnostics, several additional follow up studies are needed to 1) replicate the findings in independent and larger samples, and 2) test and validate the approach in populations for whom this tool would be most useful clinically. These populations include the following: 1) those with early stages of illness, namely, adolescents who have not yet experienced significant weight loss and stand to benefit from targeted interventions that could prevent the most impactful manifestations of the illness; 2) those at risk of developing either disorder, perhaps because of a strong family history and/or anxious temperament in childhood; and 3) those who fall on the border between AN and BDD, as exemplified in the Introduction.

Additional testing and validation in acute, rather than weight-restored, AN populations is necessary to understand its generalizability. A strong caveat of the current dataset, therefore, is that the informative neurobiological feature of NPL, and conceivably even insight, may have been influenced by previous starvation states in AN participants. It is necessary to determine the utility of this classification procedure using the current features through future validation, which can be accomplished through the longitudinal study of individuals at risk or in very early pre-starvation stages of symptom manifestation. Similarly, testing in normative adolescent populations is also necessary given the more nuanced differences in appearance concerns and behaviors between healthy adolescents and individuals who meet specific clinical criteria. Additionally, given the increased likelihood of acquisition heterogeneity in multimodal data, these results must be tested for generalizability across worldwide research centers.

If validated successfully, our multimodal, two-stage classification model may aid in clinical disambiguation between healthy individuals and individuals with AN or BDD. Critically, if the methodology proves accurate in populations that are at risk or very early in the course of illness, then it may contribute to the prevention of the starvation state. This step not only would help reduce the risk of medical morbidity and mortality, but also could improve the effectiveness of other treatments that have difficulty taking hold in the context of extreme malnutrition [61,62].

## Supporting information

S1 File Detailed methods. Additional information regarding fMRI methods, imputations, and statistical modeling.
(PDF)
