# Supplementary material for: Differentiating weight-restored anorexia nervosa and body dysmorphic disorder using neuroimaging and psychometric markers
Source: PLoS One. 2019 May 6;14(5):e0213974. doi: 10.1371/journal.pone.0213974 (PMC6502309; doi:10.1371/journal.pone.0213974)
Supplement: S1 File — Additional information regarding fMRI methods, imputations, and statistical modeling. (PDF) [file pone.0213974.s001.pdf]

# Supplementary material:
## *Differentiating weight-restored anorexia nervosa and body dysmorphic disorder using neuroimaging and psychometric markers*

**fMRI data, continued:**

We acquired magnetic resonance (MR) images using a Siemens Trio 3T scanner equipped with a 12-channel head coil. We acquired T1-weighted images with an MP-RAGE sequence and blood oxygenation level dependent (BOLD) signal changes from task-based fMRI using body visual stimuli.

We preprocessed participant data with high-pass temporal filtering and nonlinear boundary-based registration, and then performed independent component analysis (ICA)-based Automatic Removal Of Motion Artifacts (ICA-AROMA) with non-aggressive de-noising (Pruim *et al*, 2015). Using FEAT (part of FMRIB's Software Library, or FSL), fMRI network coherence analysis was carried out in a two-stage process using Multivariate Exploratory Linear Optimized Decomposition into Independent Components (MELODIC) (Beckmann and Smith, 2004).

In stage 1, MELODIC was used to identify large-scale connectivity patterns in all participants. A group-level ICA was performed with the de-noised single participant data as inputs, resulting in a decomposition of our data set into 20 independent components. These group ICA components, specific for when participants viewed high spatial-frequency bodies, were then correlated with canonical ICA networks as previously described (Smith *et al*, 2009). The components with the highest correlations with canonical networks for our networks of interest were used to select our network-specific masks.

In stage 2, we identified subject-specific temporal dynamics and associated spatial maps for each subject's fMRI data. This step used the full set of group-ICA spatial maps in a spatial regression for a linear fit against the separate fMRI data sets. The resulting time-course matrices (comprised of the temporal dynamics for each component and each subject) were then used in a temporal regression for a linear model fit against the fMRI data to estimate subject-specific *spatial* maps. The spatial maps from stage 2 were used to calculate coherence values for each subject within each network mask.

**Handling of missing data, continued:**

We accomplished multiple imputation by creating 20 independent filled-in (imputed) datasets, and then analyzing each separate dataset. This technique supposes that data is missing completely at random, which is defined by no relation between the probability of missingness and the value of the missing data point. The results from each dataset were combined according to the between and within imputation variation (Rubin, 2004). We initiated each

dataset with cold-deck imputation: the missing values were filled in randomly, with replacement, from the other observed values. To improve upon these uninformed estimates, we trained a linear regression model to predict the mean and variance of each missing value based on other observed variables (e.g., MH and NPL), not considering the diagnosis of any participants. The missing values were filled in stochastically, based on the predicted mean and variance. A coordinate descent method was used to impute multiple variables where, when imputing missing BABS values, the most recent iteration of imputed coherence values were used to train the imputation model, and vice-versa for imputing coherence based on BABS. This was completed for over 1,000 iterations. For the first 100 cycles, we artificially multiplied the variance by an exponentially decaying factor such that the variance increased by 1% after 100 cycles; this provided a more complete exploration of the likelihood space of imputation models. After roughly 150 cycles, the deviance of the imputation models had reached an equilibrium. We used residual deviance from prediction as our goodness of fit measure because for least-squares residuals, minimization of deviance results in the maximum likelihood estimate of statistics.

### Diagnostic Statistical Modeling, continued:

In leave-one-out training, the predictive model is trained excluding one participant, then validated using the excluded participant. The identity of that excluded participant is then changed so that each participant is the validation participant once and only once.

We evaluated model performance by comparing our observed results to those of identical models trained on 10,000 independent datasets in which the participant diagnoses were shuffled without replacement. The permuted results were used to create empirical probability distributions of each reported statistic, yielding an empirical, two-tailed p-value indicated by $\widehat{p}$ . Permutation tests were used to avoid distributional assumptions of reported statistics that could have been made inaccurate by multiple imputation and other factors.

### Author Contributions

JDF designed the experiment. JDF and TDM collected the data. DAV, TDM, Aifeng Zhang, Alex Leow, Michael Strober, and GKC processed the data. DAV and WTK performed the classification analysis under the guidance of MSC. DAV, WTK, and JDF wrote the manuscript, with editorial contributions from MSC.

### References

Beckmann CF, Smith SM (2004). Probabilistic independent component analysis for functional magnetic resonance imaging. *IEEE Trans Med Imaging* **23**: 137–152.

Pruim RHR, Mennes M, Rooij D van, Llera A, Buitelaar JK, Beckmann CF (2015). ICA-AROMA: A robust ICA-based strategy for removing motion artifacts from fMRI data. *Neuroimage* **112**: 267–277.

Rubin DB (John Wiley & Sons: 2004). *Multiple Imputation for Nonresponse in Surveys*. .

Smith SM, Fox PT, Miller KL, Glahn DC, Fox PM, Mackay CE, *et al* (2009). Correspondence of the brain's functional architecture during activation and rest. *Proceedings of the National Academy of Sciences* **106**: 13040–13045.
